# Salt-Responsive Transcriptome Profiling of *Suaeda glauca* via RNA Sequencing

**DOI:** 10.1371/journal.pone.0150504

**Published:** 2016-03-01

**Authors:** Hangxia Jin, Dekun Dong, Qinghua Yang, Danhua Zhu

**Affiliations:** Zhejiang Academy of Agricultural Science, Institute of Crops and Nuclear Technology Utilization, Hangzhou Zhejiang 310021, People’s Republic of China; University of Western Sydney, AUSTRALIA

## Abstract

**Background:**

*Suaeda glauca*, a succulent halophyte of the Chenopodiaceae family, is widely distributed in coastal areas of China. *Suaeda glauca* is highly resistant to salt and alkali stresses. In the present study, the salt-responsive transcriptome of *Suaeda glauca* was analyzed to identify genes involved in salt tolerance and study halophilic mechanisms in this halophyte.

**Results:**

Illumina HiSeq 2500 was used to sequence cDNA libraries from salt-treated and control samples with three replicates each treatment. *De novo* assembly of the six transcriptomes identified 75,445 unigenes. A total of 23,901 (31.68%) unigenes were annotated. Compared with transcriptomes from the three salt-treated and three salt-free samples, 231 differentially expressed genes (DEGs) were detected (including 130 up-regulated genes and 101 down-regulated genes), and 195 unigenes were functionally annotated. Based on the Gene Ontology (GO), Clusters of Orthologous Groups (COG) and Kyoto Encyclopedia of Genes and Genomes (KEGG) classifications of the DEGs, more attention should be paid to transcripts associated with signal transduction, transporters, the cell wall and growth, defense metabolism and transcription factors involved in salt tolerance.

**Conclusions:**

This report provides a genome-wide transcriptional analysis of a halophyte, *Suaeda glauca*, under salt stress. Further studies of the genetic basis of salt tolerance in halophytes are warranted.

## Introduction

Soil salinization restricts plant growth and reduces crop yields, motivating interest in the mechanisms of regulation of salt tolerance in plants. Most crops cannot survive or produce biomass in salty soils, whereas halophytes can grow normally in heavily saline soils [1, 2]. The salt tolerance of halophytes may be attributed to multiple regulatory mechanisms [3–5]. A few genes related to the salt tolerance of halophytes have been cloned, but the mechanisms by which halophytes survive in high-salinity soils remains unclear. The elucidation of differences in salt-tolerance mechanisms among halophytes and the genes that play major roles in these mechanisms have been limited by the lack of information on the genomes and transcriptomes of various halophytes. Next-generation sequencing has enabled genome-wide scale and transcriptome-level computational analyses. RNA sequencing (RNA-Seq) technology is an economical and efficient platform for analyzing the expression of various genes at the transcriptome level, particularly in species that do not have reference genomes [6], such as most halophytes.

*Suaeda glauca* (Bunge), a succulent obligate halophyte of the Chenopodiaceae family, is widely distributed in coastal areas of China. *Suaeda glauca* is an annual herb that is used as forage for domestic animals or as a wild vegetable and medicinal material for humans in China [7, 8]. *Suaeda glauca* exhibits high resistance to salt and alkali stresses and grows well with salt content >0.48% [9], even without salt glands and bladders in its leaves. Under salt stress, *Suaeda glauca* accumulates organic acids and inorganic anions to maintain the intracellular ionic equilibrium, specially compartmentalizes excess Na^+^ into vacuoles of mesophyll cells [10, 11]. However, other mechanisms underlying the salt tolerance of *Suaeda glauca* remain unknown. A global transcriptome analysis of salt treated *Suaeda glauca* will help us a lot on the understanding of salt-tolerant machanisms.

In this study, the seedlings of *Suaeda glauca* didn’t show symptoms of salinity injury after imposing 100mM-300 mM NaCl stress. RNA-seq was performed to examine the transcriptomes of shoot samples of the salt-treated or control plants. A total of 231 unigenes were induced or repressed under 300 mM salt treatment, suggesting that these genes are relevant to the salt response and tolerance. Those genes were further explicated and discussed in this paper.

## Materials and Methods

### Plant materials and salt stress treatment

*Suaeda glauca* (Bunge) seeds were collected from coastal saline-alkali soil in Cixi County, Zhejiang Province, Southeast China at 121.21°/30.26° (longitude/latitude). No specific permits were required for plant collection in this study and all plant specimens were obtained from public, not owned territory; therefore, no specific permissions were required for seeds collection. Collecting seeds of *Suaeda glauca* in that area did not involve endangered or protected species. Seeds were planted on vermiculite damped with water and grew under 25°C in a climate chamber with 16:8 hour light-dark cycle, at the Zhejiang Academy of Agricultural Science, Hangzhou, China. One-month-old seedlings were treated with 300 mM NaCl and the same volume of water (as control), with three replicates. The shoots of three NaCl-treated seedlings and three control seedlings were sampled and stored in liquid nitrogen for RNA extraction after 24 hours of salt treatment.

To assess the effects of salinity stress on *Suaeda glauca* seedlings, one-month-old seedlings were treated with 0, 100, 200, 300, 400 or 1000 mM NaCl solution dissolved in water every other day under 25°C in a climate chamber with 16:8 hour light-dark cycle. The plant height was measured on 0, 5 and 11 days after treatment.

### Quantification of K^+^ and Na^+^ content of seedlings after treatment

Shoot samples were harvested from the seedlings treated with 300 mM or 1 M NaCl for 0 h and 24 hours, and dried at 80°C to constant weight in an oven. Then the dried tissues were ground into fine powder. Tissue powders (0.1g) were mixed with 10 mL HNO_3_ (8 M) and incubated at 150°C for 6 h. Three biologically independent replicates were prepared. Then, K^+^ and Na^+^ concentrations were measured using an atomic absorption spectrophotometer (AA240; Varian Medical Systems, USA).

### RNA extraction, cDNA library preparation and sequencing

Total RNA for Illumina sequencing was isolated from shoot tissues of plants grown under salt treatment or control conditions using a Quick RNA Isolation Kit (BioTeke Corporation, Beijing, China). The quantity and quality of the total RNA were assessed using a NanoDrop ND1000 spectrophotometer (NanoDrop Technologies, Wilmington, DE, USA), Qubit 2.0 fluorometer (Life Technologies, Carlsbad, CA, USA) and an Agilent 2100 Bioanalyzer (Santa Clara, CA, USA). The cDNA library was constructed and sequenced by the Biomarker Biotechnology Corporation (Beijing, China). The poly (A) mRNA was enriched via magnetic oligo (dT) beads and then broken into short fragments using an RNA Fragmentation Kit (Beckman Coulter, Brea, CA, USA). These cleaved mRNA fragments were used as templates for first-strand cDNA synthesis using random hexamer primers. Then, second-strand cDNA was synthesized and purified using AMPure XP beads (Beckman Coulter, Brea, CA, USA). These short fragments were ligated to sequencing adapters, and the desired fragments were separated using AMPure XP beads. Next, the purified cDNA fragments were enriched via PCR. Finally, the six cDNA libraries were sequenced using Illumina HiSeq^™^ 2500 and 125bp paired-end reads were generated.

### Sequence assembly and annotation

The raw reads were filtered by removing low-quality sequences and rRNA reads. Then, *de novo* assembly of the clean reads was performed using the Trinity [12] platform (http://trinityrnaseq.sourceforge.net/). Short reads were first assembled to form the longest contigs according to their overlapping regions. These contigs were clustered into components, and Bruijn graphs were constructed. Finally, the sequences that could not be elongated at each terminus were defined as unigenes. All unigenes were analyzed via sequence alignment with the NR (NCBI non-redundant protein), Swiss-Prot, GO (Gene Ontology), COG (Clusters of Orthologous Groups) and KEGG (Kyoto Encyclopedia of Genes and Genomes) databases using BLAST2GO [13].

### Identification of differentially expressed genes

To identify genes that were differentially expressed between the salt-treated and salt-free samples, gene expression-level analysis was performed using the FPKM [14] (Fragments Per Kilobase of transcript per Million mapped reads) method. To compute the significance of the differences in gene expression levels, an FDR (false discovery rate) method was used to determine the threshold of the P-value. Then, the genes potentially regulated by salt stress were identified using an FDR threshold < 0.01, p value < 0.001 and fold change ≥ 2 (log2-fold change (FC) ≥ 1 or log2 FC ≤ -1) between all three salt-treated and three salt-free samples using DESeq software [15].

### QPCR analysis

Total RNA was extracted from the shoot tissues of the plant samples. First-strand cDNA was synthesized using oligo (dT) primers. Six unigenes were subjected to real-time quantitative PCR (qPCR) with specific primers (S4 Table) designed based on the sequences of the contigs. The internal reference gene was the *actin* gene. QPCR analysis of the ten genes was performed in triplicate with SYBR Green Real-time PCR Master Mix (TOYOBO, Osaka, Japan) on a LightCycler^®^ 480 Real-Time PCR System (Roche Diagnostics GmbH, Mannheim, Germany).

## Results and Discussion

### Salinity tolerance of *Suaeda glauca*

**Fig 1:**
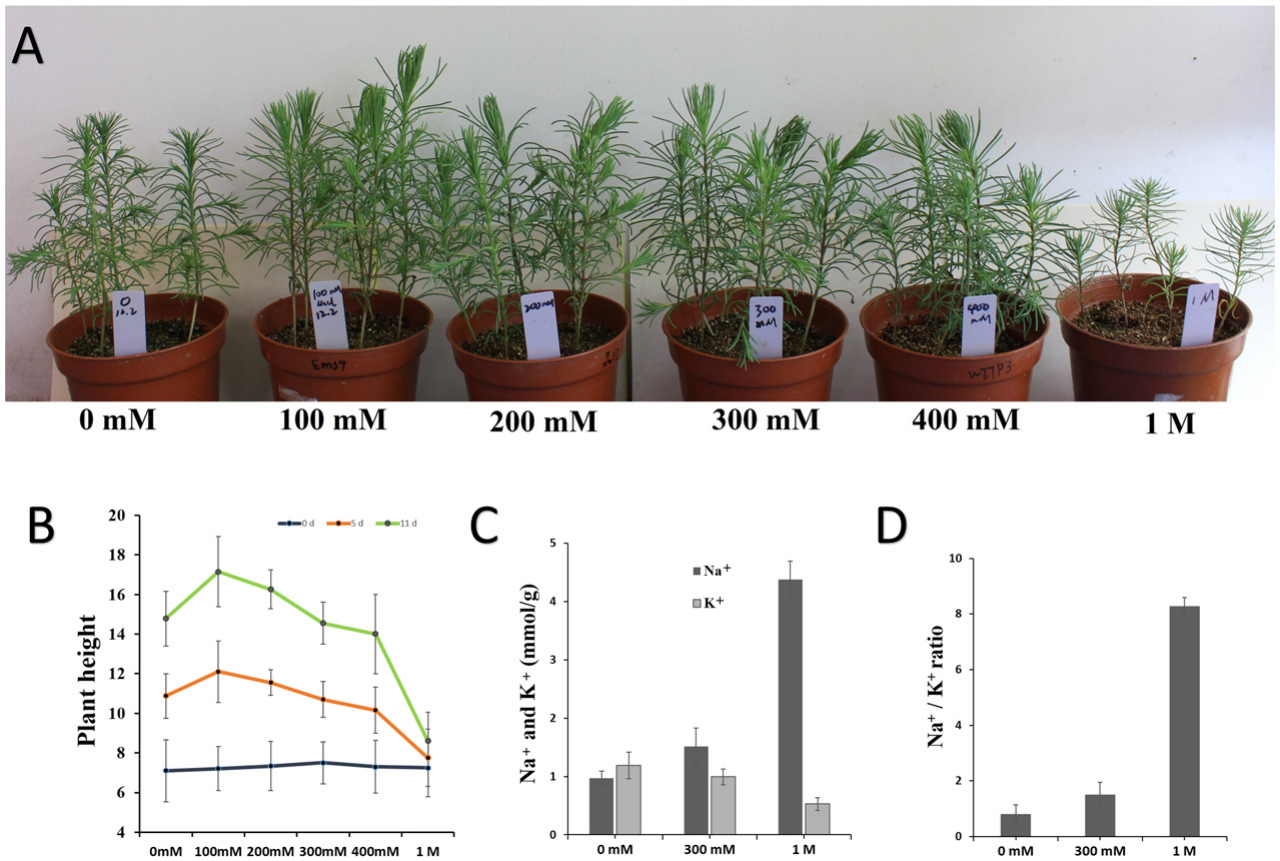
Responses of *Suaeda glauca* to salinity stress. (A) Differential responses of seedlings to salinity stress after 11 days. The NaCl concentrations are indicated in the white text boxes (0, 100, 200, 300, 400 mM and 1 M). (B) Effects of different NaCl concentrations on the mean plant height of *Suaeda glauca* seedlings on 0, 5 and 11 days. The x-axis represents different NaCl concentrations, and the y-axis represents the mean plant height. (C) The Na^+^ and K^+^ contents of *Suaeda glauca* seedlings under 300 mM NaCl and 1M NaCl. (D) The Na^+^/ K^+^ ratios of *Suaeda glauca* seedlings under 300 mM NaCl and 1M NaCl.

The seedlings of *Suaeda glauca* didn’t show symptoms of salinity injury after imposing 100mM-300 mM NaCl stress (Fig 1A and 1B) compared with control (0 mM NaCl). The seedlings under 100 mM NaCl treatment showed the highest plant height (*P* < 0.05), suggesting that low concentration of NaCl might have promoting effect on the growth of *Suaeda glauca* (Fig 1B). The seedlings was severely suppressed under 1M NaCl stress (*P* < 0.001), however they survived under such severe conditions (Fig 1B).

The Na^+^ content slightly increased after 300 mM NaCl treated for 24 h, and increased significantly (*P* < 0.01) after 1 M NaCl treated for 24 h (Fig 1C). While the K^+^ content slightly decreased after 300 mM NaCl treated for 24 h, and decreased significantly (*P* < 0.001) after 1 M NaCl treated for 24 h. The changing trends of Na^+^/ K^+^ ratios were similar with that of the Na^+^ content (Fig 1D). It seems that a large amount of Na^+^ accumulated in shoot tissues which might repress plant growth under 1 M NaCl, while the Na^+^ and K^+^ contents showed a slight change under 300 mM NaCl compared with normal condition.

### RNA-Seq and assembly

**Fig 2:**
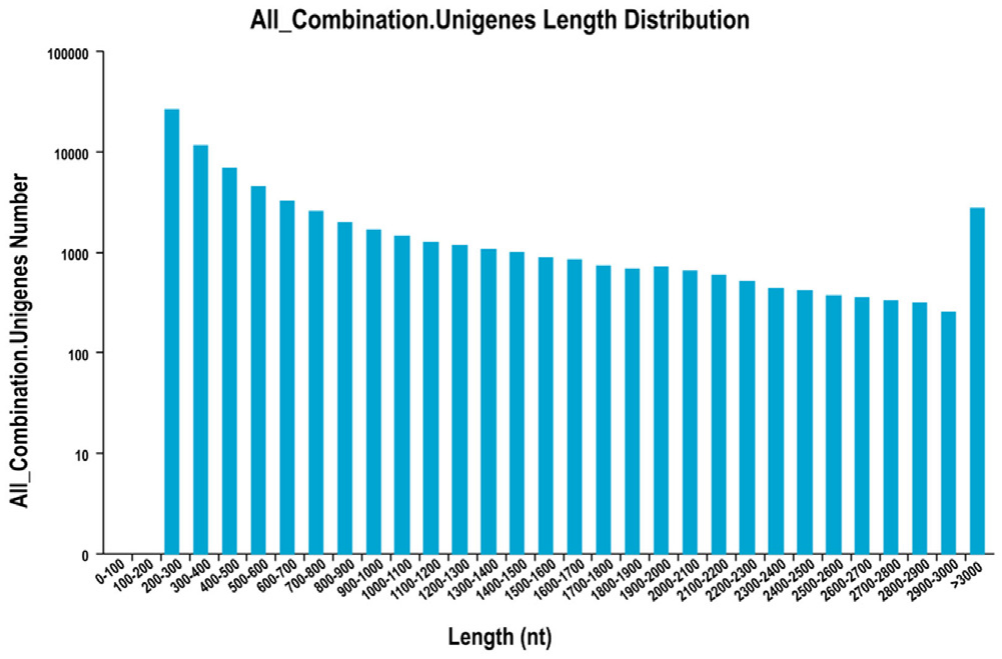
Size distribution of the unigenes generated via de novo assembly. The x-axis represents the All-Unigenes lengths, and the y-axis represents the number of All-Unigenes in a certain length range.

To obtain a more comprehensive understanding of the salt-induced changes at the transcriptome level in *Suaeda glauca*, six cDNA libraries (salt-treated and control, three replicates) were sequenced. A total of 45.17 Gb of clean data were obtained; each sample yielded up to 6.8 Gb of clean data and a Q30 percentage above 91.03% (Table 1). The reads from six libraries were combined and assembled into 75,445 unigenes using the Trinity method. The N50 length of the *Suaeda glauca* contigs was 1,425 bp. A total of 16,765 unigenes were longer than 1 kb and 10,792 were longer than 1.5 kb in length, indicating that nearly full-length transcripts were obtained for some candidate genes (Fig 2).

**Table 1:** Total number of reads for each sample.

Sample	Raw Read	Clean reads	Use Data(G)
T1	27847578	27135313	6.836311
T2	29353684	28570354	7.198305
T3	33203005	32074265	8.08093
T4	34606210	33426317	8.421403
T5	29852794	29021465	7.311863
T6	29963090	29062110	7.321849

### Sequence annotation of all unigenes and the differentially expressed genes

Sequence alignments to the NR, Swiss-Prot, GO, COG and KEGG databases revealed that of the 75,445 high-quality unigene sequences from *Suaeda glauca*, only 23,901 unigenes (31.68%) were significantly matched to known genes (S1 Table).

**Fig 3:**
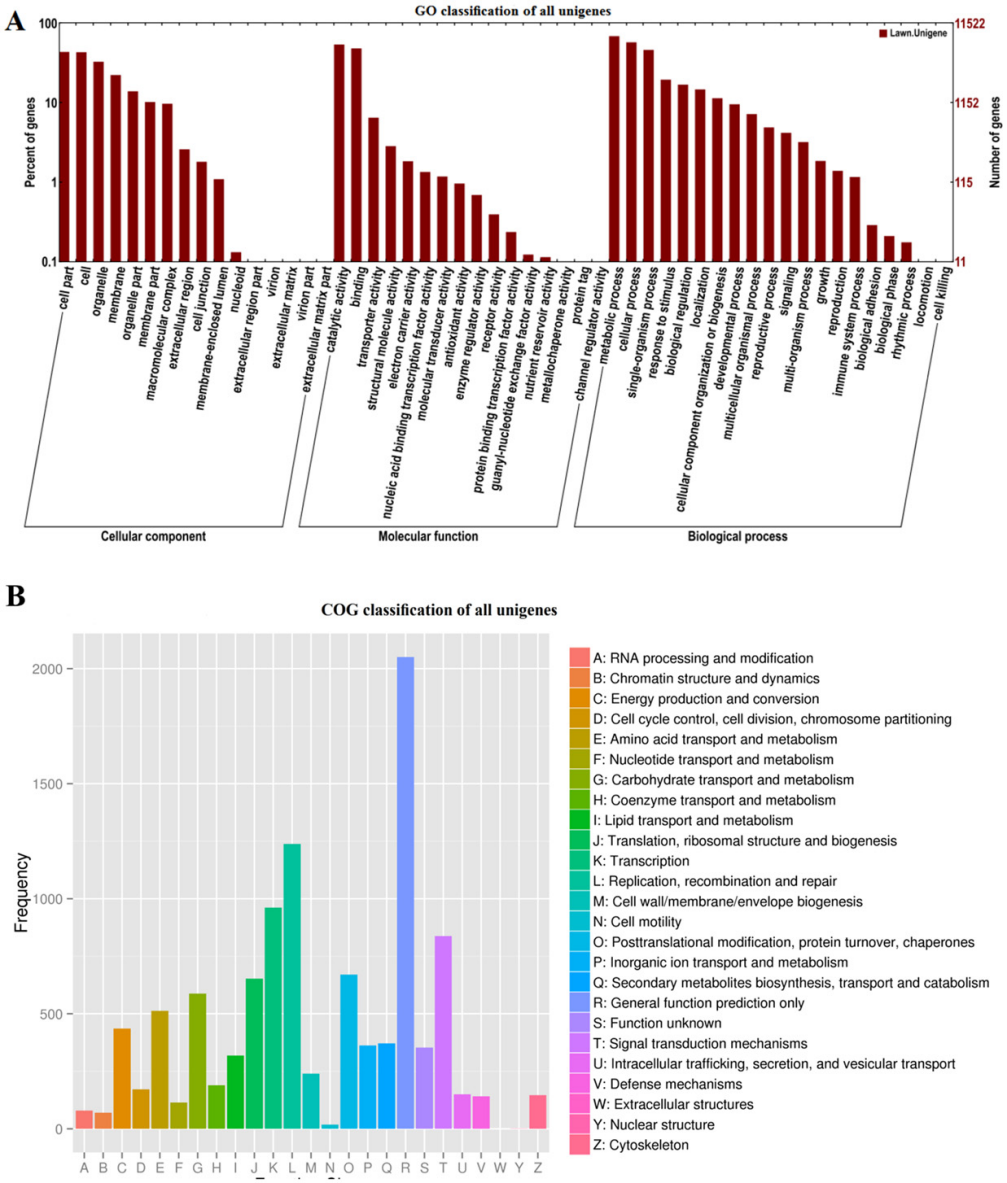
GO classfication and COG classification of all unigenes. (A) All unigene sequences with BLAST2GO matches were assigned to three GO categories (cellular component, molecular function, and biological process) and classified into 41 functional terms. (B) Sequences with BLAST2GO matches were assigned to the COG database and classified into 24 functional categories.

Among the functional categories of the 23,901 unigenes, a total of 11,522 (48.21%) were assigned at least one GO term and were classified into different functional terms from three GO categories (Fig 3A). A total of 10,668 (44.63%) unigenes were aligned to the COG database and classified into 24 functional categories (Fig 3B). Only 4,457 (18.65%) unigenes aligned with the KEGG database and were assigned to 116 KEGG pathways (S1 Table).

To further predict the differentially expressed genes (DEGs) under salt-stressed conditions, GO and COG functional analyses were performed. DEGs were defined according to a threshold FDR < 0.01 and fold change ≥ 2 (log_2_-fold change (FC) ≥ 1 or log_2_ FC ≤ −1) between the three salt-treated and three salt-free samples.

**Fig 4:**
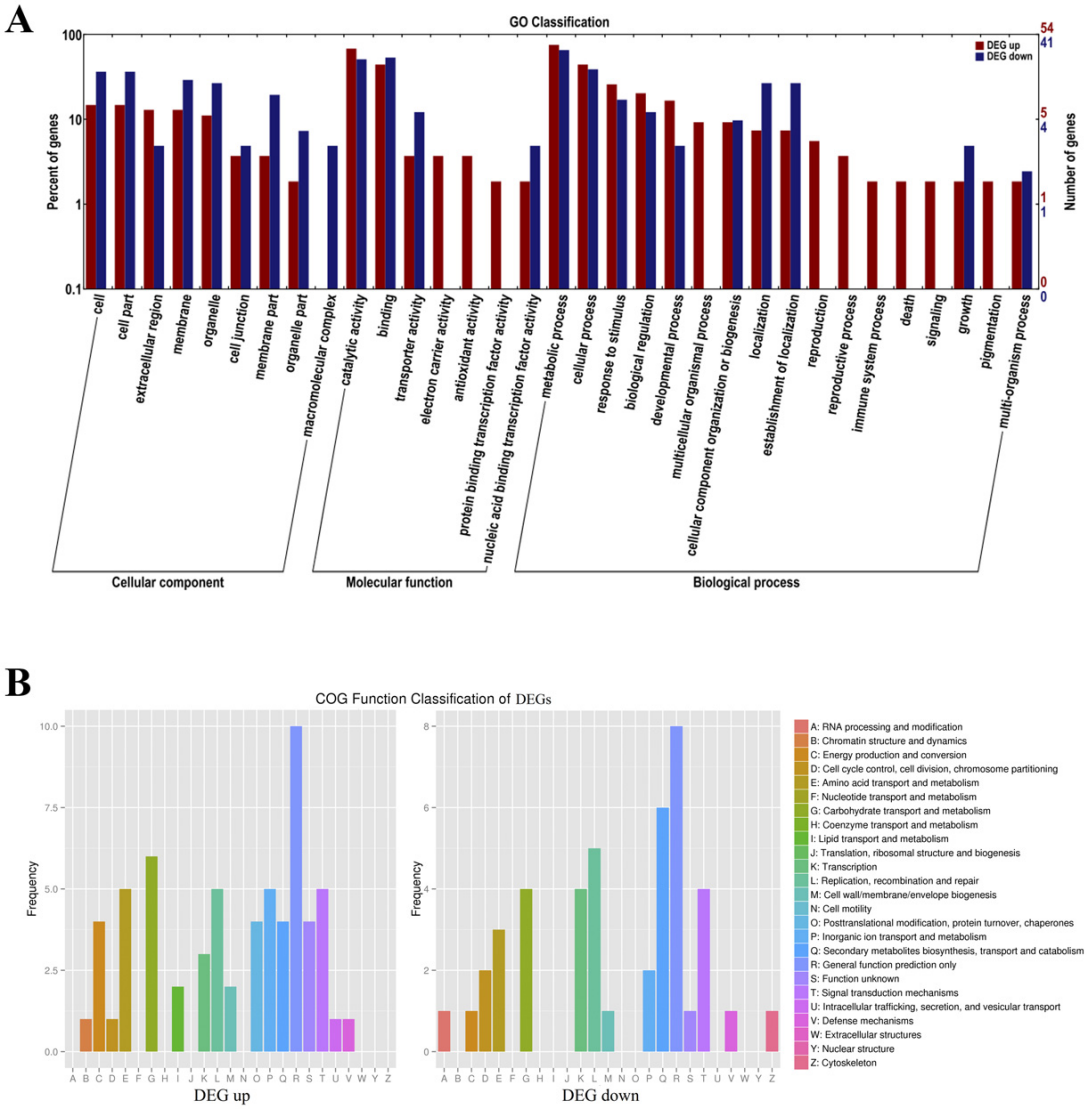
GO classification and COG classification of DEGs. (A) 54 up-regulated genes and 41 down-regulated genes of DEGs were assigned to three GO categories and classified into 33 functional terms. (B) 42 up-regulated genes and 30 down-regulated genes of DEGs were assigned to the COG database and classified into 20 functional categories.

A total of 231 DEGs were detected, including 130 up-regulated genes and 101 down-regulated genes, and 195 unigenes were functionally annotated (S2 Table). GO annotation analysis classified 95 DEGs into three GO categories and 32 terms (Fig 4A). In the molecular function category, 78 DEGs were classified into seven terms, including protein binding transcription factor activity, nucleic acid binding, transcription factor activity, catalytic activity, transporter activity, binding electron carrier activity and antioxidant activity. To further clarify molecular function, 78 DEGs were enriched in 84 terms. COG and KOG annotation analyses revealed that 105 DEGs were functionally annotated, 42 up-regulated genes and 30 down-regulated genes of DEGs were assigned to the COG database and classified into 17 and 15 functional categories, respectively (Fig 4B and S3 Table). The top hits included inorganic ion transport and metabolism (9.52%), lipid transport and metabolism (6.67%), cell wall metabolism (5.63%), transcription factors (5.2%), transporters (3.9%), signal transduction mechanisms (3.9%) and defense mechanisms (3.9%).

### Validation of DEGs by real-time quantitative PCR

**Fig 5:**
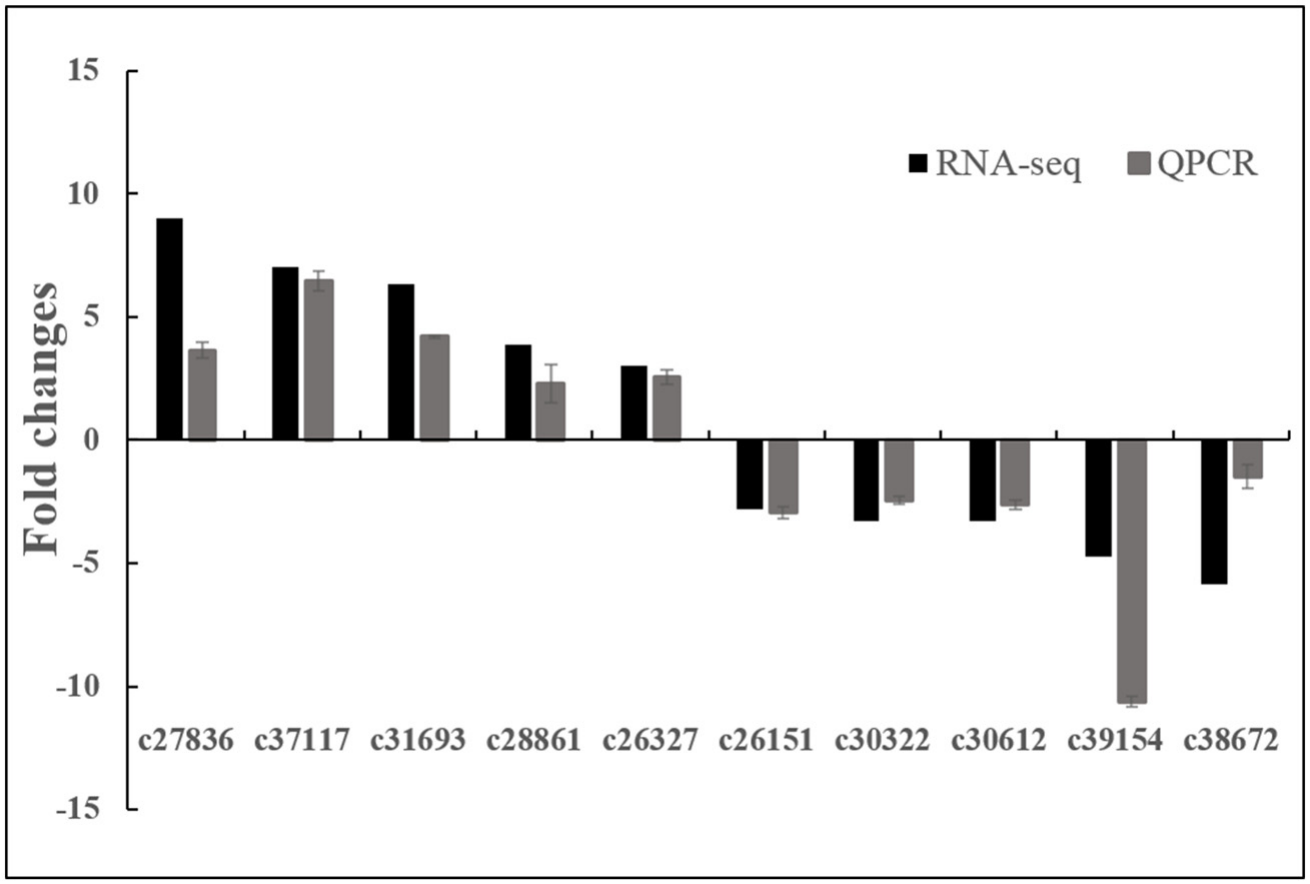
Verification of RNA-seq results by real-time quantitative PCR (QPCR). The black bars represent fold changes based on FPKM calculated from globally normalized RNA-seq data. The gray bars with standard errors indicate fold changes based on the relative expression level determined by qPCR using the 2^-ΔΔCT^ method for three biological replicates under saline (300 mM NaCl) and normal conditions.

The salinity-responsive DEGs identified from the RNA-Seq data were confirmed by real-time quantitative PCR (qPCR). Five up-regulated DEGs and Five down-regulated DEGs under salinity were selected. Good agreement between qPCR data and RNA-seq data was shown by Pearson correlation analysis of the fold change measurements (R^2^ = 0.84, *P* = 0.002) (Fig 5).

### Detection of salt-induced genes

*Suaeda glauca* is a succulent halophyte that can survive in saline-alkaline soils [8, 9]. This plant has high salt tolerance and consequently represents an excellent resource for studying the mechanism of salinity tolerance. This halophyte may thus provide a valuable source for validating salinity tolerance-related genes.

Analysis of the transcriptome for salt tolerance in *Suaeda glauca* identified 231 DEGs. Among the DEGs, about 20% genes assembled remarkably to five functional categories, including signal transduction, cell wall metabolism, defense metabolism, transporters and transcription factors, which may have specific biological function in salt-tolerant mechanism of *Suaeda glauca*. There were also 36 DEGs having no function annotation and may play unique roles in stress adaptation by *Suaeda glauca*.

#### Signal transduction

Signal transduction is important for plant adaptation to environmental stresses, including salinity stress [16–19]. In the present study, genes involved in signal transduction mechanisms were differentially expressed under salinity stress. The genes encoding protein phosphatase 2C 24 (c37117.graph_c0) and protein phosphatase 2C 8 (c26998.graph_c0) were up-regulated in response to salinity. These phosphatases are key regulators of ABA signaling that function both negatively [20, 21] and positively to enhance plant abiotic tolerance. For instance, the over-expression of AtPP2CG1 (Arabidopsis thaliana protein phosphatase 2C G Group 1) leads to enhanced salt tolerance, whereas its loss of function reduces salt tolerance [22]; over-expression of ZmPP2C2 in tobacco enhances tolerance to cold stress [23]. Similar studies were performed *in Suaeda fruticosa*, a halophyte species closely related to *Suaeda glauca*, which found that phosphatase 2C family proteins were upregulated at 300 mM NaCl treatment. Therefore, protein phosphatase 2C may play an important role in *Suaeda glauca* salinity tolerance similar with its congeneric specie *Suaeda fruticosa*.

Abscisic acid (ABA) is a major endogenous signal. Abiotic stress conditions such as salinity induce ABA biosynthesis, which activates signaling pathways that lead to a series of responses [24]. ABA 8'-hydroxylase, a cytochrome P450, is thought to play a dominant role in ABA catabolism [25], whereas other cytochrome P450s may mediate growth and stress responses [26, 27]. In the present study, three unigenes encoding cytochrome P450s (c38030.graph_c0, c36311.graph_c0, c35237.graph_c0) were up-regulated, and one gene encoding cytochrome P450 71A4 (c11039.graph_c0) and two unigenes encoding cytochrome P450 86B1 (c40560.graph_c0, c26976.graph_c0) were down-regulated. But the detailed function of each cytochrome P450 in *Suaeda glauca* is still unknown.

Interestingly, the above genes may all be related to ABA signal transduction, suggesting ABA signal transduction may play an important role and reflect timely in the earlier stage when suffering from salt stress.

#### Transporters

Oligopeptide transporters (OPTs) are membrane-localized proteins with a broad range of substrate transport capabilities, including glutathione [28] and metal transport [29–31]. In this study, one gene encoding an oligopeptide transporter (c36906.graph_c0) was identified as up-regulated, indicating that this gene may be related to metal transport and homeostasis under salinity.

ATP-binding cassette (ABC) proteins transport a wide range of molecules across membranes including hormones, lipids, metals, secondary metabolites and modulators of ion channels [32, 33]. ABC transporters enhance salt and drought resistance [34] or transport ABA in Arabidopsis [33]. Here, a homologue (c38340.graph_c0) of an ABC transporter from *Arabidopsis thaliana* was up-regulated, suggesting that this gene may have a similar function in *Suaeda glauca*.

To control ionic homeostasis is an important mechanism of salinity tolerance. The Na^+^ influx transporter (HKT) and the tonoplast Na^+^/H^+^ antiporter (NHX) are reported to be involved in Na^+^ homeostasis and vacuolar compartmentation under salt stress in plants. However, HKT and NHX antiporter didn’t exist significantly different expression between salt-treated and salt-free samples in *Suaeda glauca*, while they were up-regulated under salt stress in other plants such as halophyte *Salicornia europaea* [35], halophyte *Atriplex lentiformis* and *Chenopodium quinoa* [36], *Populus euphratica* [37] and chickpea [38]. A similar trend appeared *in Suaeda fruticosa*, even one HKT1-like transporter of *Suaeda fruticosa* was downregulated under salt stress. So, different from other plants, *Suaeda* specises may have some similar and particular pathways to adapt saline condition. Perhaps the basal expression level of such antiporter including HKT and NHX is enough to cope with salt stress at the concentration of 300 mM NaCl in *Suaeda glauca*.

#### Cell wall and growth

Salt stress disturbs the normal growth and development of plants. Modification of the cell wall is a common defense response when plant suffering from abiotic and biotic stress. In our study, most of genes related to cell wall and growth were up-regulated under salt stress.

Expansin-A4 (c20751.graph_c0) and expansin-A2 (c36645.graph_c0), which are involved in cell elongation and cell wall modification by loosening and extending plant cell walls [39], were up-regulated in salt-treated samples.

Leucine-rich repeat extensins (LRX) involving in cell wall assembly are potential regulators of cell wall development in Arabidopsis [40]. Here, a homologue (c38524.graph_c0) of LPX from *Arabidopsis thaliana* and a homologue (c33977.graph_c0) of LPX from *Beta vulgaris* were up-regulated.

Wall-associated receptor kinase proteins which link the plasma membrane to the extracellular matrix have been implicated in cell elongation, plant responses to pathogens [41, 42]. In *Suaeda glauca*, one unigene c40146.graph_c1 encoding wall-associated receptor kinase protein was up-regulated.

The O-acyltransferase WSD1 plays a key role in wax ester synthesis in Arabidopsis (*Arabidopsis thaliana*) stems [43]. Two unigenes encoding O-acyltransferase WSD1 (c30527.graph_c0 and c20079.graph_c0) were up-regulated.

These results suggested that cell wall growth could be promoted at the earlier stage of 300mM NaCl treatment, which was consistent with our observation that the seedlings of *Suaeda glauca* didn’t show symptoms of salinity injury after imposing 300mM NaCl stress (Fig 1C) compared with normal condition. It might be one of the cell adaptation mechanisms to salt in *Suaeda glauca*.

#### Defense metabolism

Salinity stress induces the production of ROS (reactive oxygen species), which cause oxidative damage in various plant cellular components. Antioxidant metabolism, including antioxidant enzymes and nonenzymatic compounds, plays an important role in detoxifying ROS. Antioxidant enzymes include ascorbate peroxidase (APX), peroxidase (POX), superoxide dismutase (SOD), catalase (CAT), and glutathione reductase (GR). In *Suaeda glauca*, the unigene c44071.graph_c0, which is a homologue of an ascorbate peroxidase from *Beta vulgaris*, and the unigene c26327.graph_c0, which is a homologue of a peroxidase from *Beta vulgaris*, were up-regulated after salt treatment. Therefore, APX and POX may be involved in ROS scavenging under salinity stress in *Suaeda glauca*.

Flavonoids have been linked to defense metabolism against various stresses, such as wounding, UV-B and salt [44, 45]. In the present study, a dihydroflavonol-4-reductase (c32549.graph_c0) involved in flavonoid metabolism [46] was up-regulated, suggesting that flavonoids may be involved in plant defense metabolism in *Suaeda glauca* under salinity stress.

Carboxylesterases are widely distributed in mammals, insects, bacteria and plants. Carboxylesterases play a role in the detoxification or metabolic activation of various drugs, carcinogens and environmental toxins in animals [47]. In Arabidopsis, isoprenylcysteine methylesterases is a carboxylesterase involved in salt stress and ABA signaling [48]. In this study, unigene c25630.graph_c0, which is a homologue of a carboxylesterase, was up-regulated and may be involved in salt tolerance.

Plant aldo-keto reductases (AKRs) are involved in biotic and abiotic stress defense, the production of commercially important secondary metabolites and other processes [49, 50]. The over-expression of an aldo-keto reductase (PpAKR1) from *Prunus persica* increased salt tolerance in Arabidopsis [51]. In this study, unigene c32029.graph_c0, a homologue of an aldo-keto reductase, was up-regulated, suggesting that this gene may be involved in salt tolerance in *Suaeda glauca*.

These results indicated defense metabolism including antioxidant enzymes and nonenzymatic compound participated in cell adaptation to salt in *Suaeda glauca*.

#### Transcription factors involved in salt tolerance

A variety of transcription factors (TFs) play important roles in resistance to abiotic and biotic stresses by regulating stress-responsive genes, such as ethylene-responsive transcription factors (ERFs) and WRKY transcription factors [52–55]. ERFs confer salt stress tolerance to plants [56–59]. Two unigenes (c12464.graph_c0 and c12432.graph_c0) encoding ERFs were up-regulated under salt stress. WRKY transcription factors are involved in drought and salt tolerance in *Gossypium hirsutum* [60], *Populus tomentosa* [61], *Jatropha curcas* [62] and *Arabidopsis thaliana* [63]. One unigene (c24234.graph_c0) encoding a WRKY transcription factor was up-regulated under salinity. Nuclear transcription factor Y (c34280.graph_c0), which is involved in salt tolerance, was also up-regulated in salt-treated samples. Over-expression of a nuclear transcription factor Y gene (*PwNF-YB3*) from *Picea wilsonii Mast* [64] or a wheat gene (TaNF-YA10-1, which belongs to the nuclear transcription factor Y family [65]) increases salt tolerance in Arabidopsis. Consistent with previous findings in other plants [66–68], high salinity induced the up-regulation of transcription factor bHLH (c28861.graph_c0). These results suggest that transcription factors may regulate salt tolerance in *Suaeda glauca*.

## Conclusions

In this report, six transcriptomes of *Suaeda glauca* under salt stress were sequenced, and 75,445 unigenes were generated with the Illumina HiSeq 2500 platform. A total of 231 DEGs were detected, including 130 up-regulated genes and 101 down-regulated genes. These DEGS included genes involved in signal transduction, transporters, cell wall and growth, defense metabolism and transcription factors involved in salt tolerance. This report provides sequence resources and a transcriptional analysis of *Suaeda glauca* that will facilitate further study of the genetic basis of the mechanism of regulation of salt tolerance in halophytes.

## Supporting Information

S1 Table Summary and functional annotation of all unigenes, including GO, COG, KOG and KEGG analysis. (XLSX)

S2 Table Summary and functional annotation of DEGs, including GO, COG, KOG and KEGG analysis. (XLSX)

S3 Table Summary of the number of DEGs assigned to different GO, COG, and KOG categories. (XLSX)

S4 Table List of primers for qPCR confirmation. (XLSX)
